# Author Correction: A micromechanics-based analytical solution for the effective thermal conductivity of composites with orthotropic matrices and interfacial thermal resistance

**DOI:** 10.1038/s41598-019-43986-x

**Published:** 2019-05-16

**Authors:** Sangryun Lee, Jinyeop Lee, Byungki Ryu, Seunghwa Ryu

**Affiliations:** 1 Department of Mechanical Engineering, Korea Advanced Institute of Science and Technology (KAIST), 291 Daehak-ro, Yuseong-gu, Daejeon 34141 Republic of Korea; 2 Department of Mathematical Sciences, Korea Advanced Institute of Science and Technology (KAIST), 291 Daehak-ro, Yuseong-gu, Daejeon 34141 Republic of Korea; 3 Thermoelectric Conversion Research Center, Korea Electrotechnology Research Institute (KERI), Changwon, Gyoengsangnam-do 51543 Republic of Korea

Correction to: *Scientific Reports* 10.1038/s41598-018-25379-8, published online 08 May 2018

The Article contains typographical errors.

Equation 4,

$${S}_{ik}({\boldsymbol{x}})=\frac{\partial }{\partial {x}_{i}}{\int }_{V}\frac{\partial G({\boldsymbol{x}}-{\boldsymbol{y}})}{\partial {y}_{j}}d{\boldsymbol{y}}{K}_{{0}_{jk}}\,{\rm{and}}\,{e}_{j}={S}_{ij}{e}_{j}^{\ast }$$

should read:

$${S}_{ik}({\boldsymbol{x}})=\frac{\partial }{\partial {x}_{i}}{\int }_{V}\frac{\partial G({\boldsymbol{x}}-{\boldsymbol{y}})}{\partial {y}_{j}}d{\boldsymbol{y}}{K}_{{0}_{jk}}\,{\rm{and}}\,{e}_{i}={S}_{ij}{e}_{j}^{\ast }$$

Equation 23,

$${\alpha }^{critical}=R(\frac{1}{\kappa }-\,\frac{1}{{k}_{I}})$$

should read:

$${\alpha }^{critical}=R(\frac{1}{{k}_{I}}-\frac{1}{\kappa })$$

