# Supplementary material for: A micromechanics-based analytical solution for the effective thermal conductivity of composites with orthotropic matrices and interfacial thermal resistance
Source: Sci Rep. 2018 May 8;8:7266. doi: 10.1038/s41598-018-25379-8 (PMC5940683; doi:10.1038/s41598-018-25379-8)
Supplement: Supplementary file 1 — Supplementary Information [file 41598_2018_25379_MOESM1_ESM.pdf]

Supplementary information

A micromechanics-based analytical solution for the effective thermal conductivity of composites with orthotropic matrices and interfacial thermal resistance

Sangryun Lee[1,†], Jinyeop Lee[2,†], Byungki Ryu[3] and Seunghwa Ryu[1,*]

Affiliations

[1]Department of Mechanical Engineering and [2]Department of Mathematical Sciences, Korea Advanced Institute of Science and Technology (KAIST), 291 Daehak-ro, Yuseong-gu, Daejeon 34141, Republic of Korea

[3]Thermoelectric Conversion Research Center, Korea Electrotechnology Research Institute (KERI), Changwon-si, Gyoengsangnam-do, 51543, Republic of Korea

[†]These authors contributed equally to this work.

[*]Corresponding author e-mail: ryush@kaist.ac.kr

Supplementary Table 1: Mathematical analogy between steady-state heat conduction, elastostatics and electrostatics.

**Heat conduction**	**Elastostatics**	**Electrostatics**
Intensity field<br>e_i $\left(e_i = -\frac{\partial T}{\partial x_i}\right)$	Elastic strain<br>ε_{ij} $\left(\varepsilon_{ij} = \frac{1}{2}\left(\frac{\partial u_j}{\partial x_i} + \frac{\partial u_i}{\partial x_j}\right)\right)$	Electric field<br>E_i $\left(E_i = -\frac{\partial V}{\partial x_i}\right)$
Temperature field<br>T	Elastic displacement<br>u_i	Electric potential<br>V
Heat flux<br>q_i	Stress tensor<br>σ_{ij}	Electric displacement<br>D_i
Fourier equation<br>$q_{i,i} = 0$	Equilibrium equation<br>$\sigma_{ij,i} = 0$	Maxwell equation<br>$D_{i,i} = 0$
Thermal conductivity tensor<br>K_{ij}	Stiffness tensor<br>L_{ijkl}	Permittivity tensor<br>ϵ_{ij}
Constitutive equation<br>$q_i = K_{ij}e_j$	Constitutive equation<br>$\sigma_{ij} = L_{ijkl}\varepsilon_{kl}$	Constitutive equation<br>$D_i = \epsilon_{ij}E_j$

Supplementary Figure 1: Eshelby tensor for anisotropic matrix

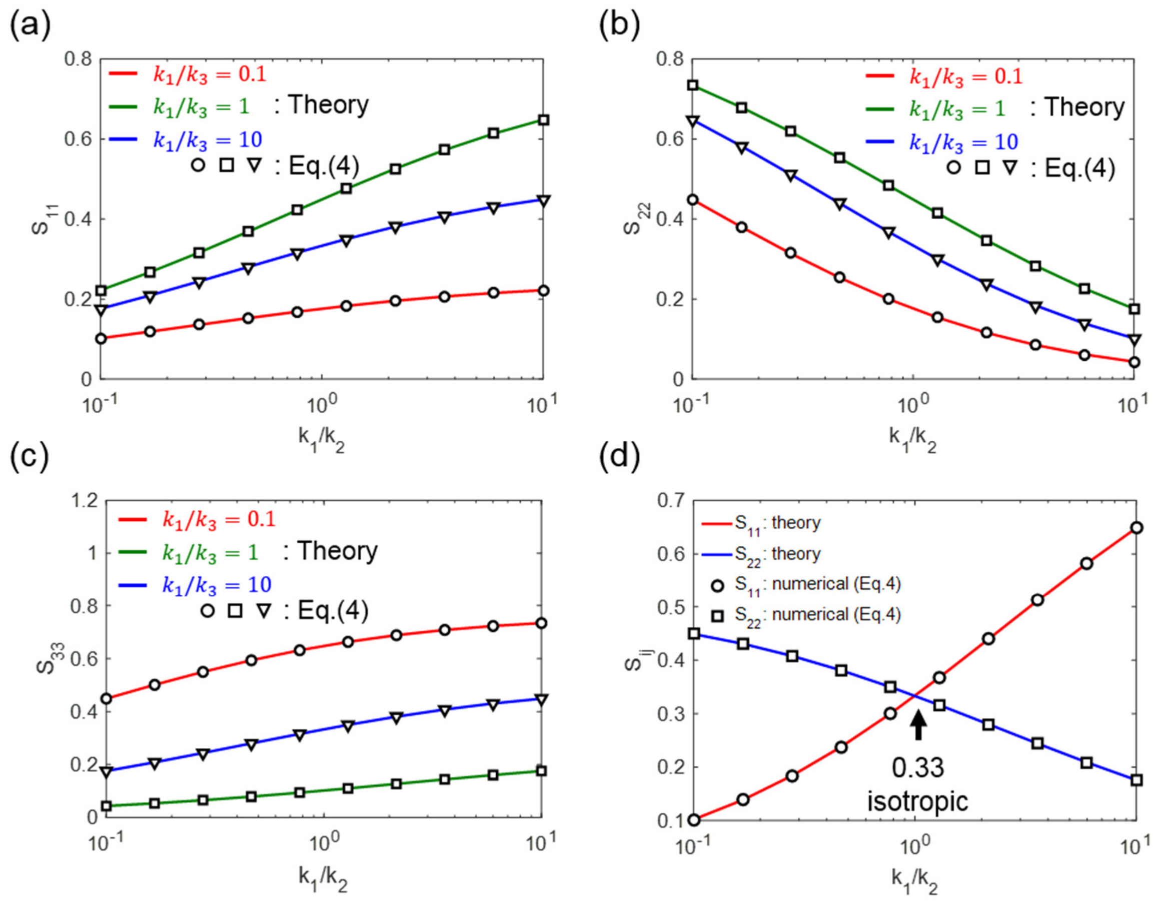

Supplementary Figure S1. The Three independent Eshelby tensor components ((a) S_{11}, (b) S_{22}, (c) S_{33}) for a spherical inclusion in an orthotropic matrix. (d) Two independent components (S_{11}, S_{22}) in the Eshelby tensor for a transversely isotropic matrix with a spherical inclusion as a function of k_1/k_2.

Supplementary Figure 2: Heat flux within ellipsoidal inclusion having interfacial thermal resistance

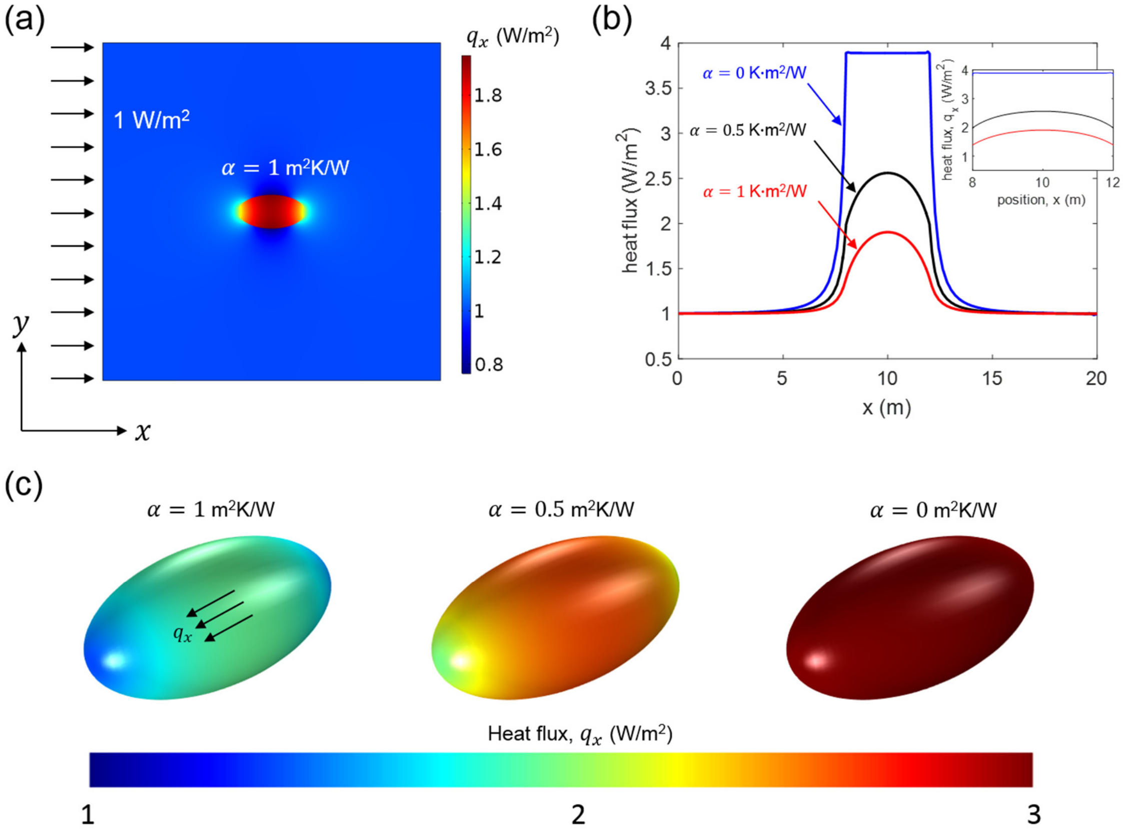

Supplementary Figure S2. (a) Heat flux in the x direction at the z=0 plane. (b) Heat flux along the long axis of inclusion (aspect ratio 2) with various interfacial resistances. (c) Heat flux at the surface of the inclusion with different Kapitza resistances. The isotropic thermal conductivities of the matrix and inclusion are 1 and 10 (W/mK) respectively, and the heat flux at the boundary is 1 W/m^2.

Supplementary Figure 3: M_{1111} component for ellipsoidal inclusion under uniform heat flux assumption

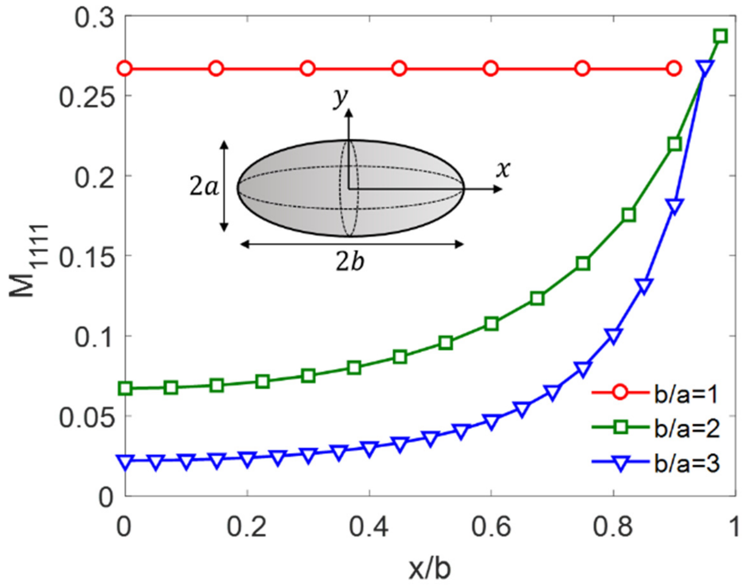

Supplementary Figure S3. M_{1111} for ellipsoids with different aspect ratios. The isotropic thermal conductivity of the inclusion is 1 W/mK.

Supplementary Note 1: Green's function for anisotropic matrix.

Comparing to Eq. (2) of previous research[1],

$$\epsilon_{kl} \frac{\partial}{\partial x_k} \frac{\partial}{\partial x_l} V(\vec{r}) = -Q(\vec{r}),$$

We use the mathematical analogy between electrostatics and heat conduction so we can formally set $k = i$, $\ell = j$, $\overleftrightarrow{\epsilon} = \boldsymbol{K}_0$, $V = G$, and $Q = 1$. Then as Eq. (3) of the previous research

$$V(\vec{r}) = \frac{1}{4\pi\sqrt{\det(\overleftrightarrow{\epsilon})\,[\vec{r}^T \overleftrightarrow{\epsilon}^{-1} \vec{r}]}}$$

obtained from Eq. (2) of the previous research, we get

$$G(\vec{r}) = \frac{1}{4\pi\sqrt{\det(K_0)\,[\vec{r}^T K_o^{-1} \vec{r}]}}$$

from

$$K_{0_{ij}} \frac{\partial}{\partial x_i} \frac{\partial}{\partial x_j} G(\vec{r}) + \delta(\vec{r}) = 0.$$

By putting $\vec{r} = \boldsymbol{x} - \boldsymbol{y}$, we get Eq. (3) from Eq. (2).

The detailed proof is in Appendix A of the previous research[1].

Supplementary Note 2: Eshelby tensor for orthotropic matrix

$$
\begin{aligned}
S_{IJ} &= \delta_{IJ}\frac{R^3}{2}\int_0^\infty \frac{(R^2+sk_I)^{-1}k_I}{\sqrt{\prod_{\ell=1}^3 (R^2+sk_\ell)}}ds \\
&= \delta_{IJ}\frac{R^3}{2}\int_0^\infty \frac{k_I}{(R^2+sk_I)\sqrt{\prod_{\ell=1}^3 (R^2+sk_\ell)}}ds \\
&= \delta_{IJ}\frac{R^3}{2}\int_0^\infty \frac{k_I}{R^2\left(1+\frac{sk_I}{R^2}\right)\sqrt{\prod_{\ell=1}^3 R^2\left(1+\frac{sk_\ell}{R^2}\right)}}ds \\
&= \delta_{IJ}\frac{R^3}{2}\int_0^\infty \frac{k_I}{R^2\left(1+\frac{sk_I}{R^2}\right)\sqrt{\prod_{\ell=1}^3 R^2\left(1+\frac{sk_\ell}{R^2}\right)}}ds \\
&= \delta_{IJ}\frac{1}{2}\int_0^\infty \frac{1}{\left(1+\frac{sk_I}{R^2}\right)\sqrt{\prod_{\ell=1}^3 \left(1+\frac{sk_\ell}{R^2}\right)}}\cdot\frac{k_I}{R^2}ds \\
&= \delta_{IJ}\frac{1}{2}\int_0^\infty \frac{1}{\left(1+\frac{sk_I}{R^2}\right)\sqrt{\prod_{\ell=1}^3 \frac{k_\ell}{k_I}\left(\frac{k_I}{k_\ell}+\frac{sk_I}{R^2}\right)}}\cdot\frac{k_I}{R^2}ds \\
&= \delta_{IJ}\frac{1}{2}\int_0^\infty \frac{1}{\left(1+\frac{sk_I}{R^2}\right)\sqrt{\prod_{\ell=1}^3 \frac{k_\ell}{k_I}}\sqrt{\prod_{\ell=1}^3 \left(\frac{k_I}{k_\ell}+\frac{sk_I}{R^2}\right)}}\cdot\frac{k_I}{R^2}ds \\
&= \delta_{IJ}\frac{1}{2}\sqrt{\prod_{\ell=1}^3 \frac{k_I}{k_\ell}}\int_0^\infty \frac{1}{\left(1+\frac{sk_I}{R^2}\right)\sqrt{\prod_{\ell=1}^3 \left(\frac{k_I}{k_\ell}+\frac{sk_I}{R^2}\right)}}\cdot\frac{k_I}{R^2}ds \\
&= \delta_{IJ}\frac{1}{2}\frac{\sqrt{\prod_{\ell=1}^3 k_I}}{\sqrt{\prod_{\ell=1}^3 k_\ell}}\int_0^\infty \frac{1}{\left(1+\frac{sk_I}{R^2}\right)\sqrt{\prod_{\ell=1}^3 \left(\frac{k_I}{k_\ell}+\frac{sk_I}{R^2}\right)}}\cdot\frac{k_I}{R^2}ds \\
&= \delta_{IJ}\frac{1}{2}\frac{k_I^{3/2}}{\sqrt{\det K_0}}\int_0^\infty \frac{1}{\left(1+\frac{sk_I}{R^2}\right)\sqrt{\prod_{\ell=1}^3 \left(\frac{k_I}{k_\ell}+\frac{sk_I}{R^2}\right)}}\cdot\frac{k_I}{R^2}ds \\
&= \delta_{IJ}\frac{1}{2}\frac{k_I^{3/2}}{\sqrt{\det K_0}}\int_0^\infty \frac{1}{(1+s')\sqrt{\prod_{\ell=1}^3 \left(\frac{k_I}{k_\ell}+s'\right)}}ds'
\end{aligned}
$$

Here we substitute the variable s as a new integral variable $s' = sk_I/R^2$ and the elliptic integral can be obtained from mathematica[2].

Supplementary Note 3: Eshelby tensor for transversely isotropic matrix

The Eshelby tensor for a particle inclusion in an orthotropic matrix with two anisotropy factors, $A = k_I/k_L$ and $B = k_I/k_M$. Suppose that $A \neq B$. Then, it can be derived as follows,

$$S_{IJ} = \delta_{IJ}\frac{(k_I)^{3/2}}{2\sqrt{\det(\boldsymbol{K}_0)}}\int_0^\infty \frac{1}{(s'+1)^{3/2}\sqrt{s'+A}\sqrt{s'+B}}ds'$$

$$= \delta_{IJ}\frac{(k_I)^{3/2}}{2\sqrt{\det(\boldsymbol{K}_0)}}\int_0^\infty \frac{1}{(s'+1)^{3/2}}\cdot\frac{1}{A-B}\left(\sqrt{\frac{s'+A}{s'+B}}-\sqrt{\frac{s'+B}{s'+A}}\right)ds'$$

$$= \delta_{IJ}\frac{(k_I)^{3/2}}{2\sqrt{\det(\boldsymbol{K}_0)}}\frac{1}{A-B}\left(\int_0^\infty \frac{\sqrt{s'+A}}{(s'+1)^{3/2}\sqrt{s'+B}}ds' - \int_0^\infty \frac{\sqrt{s'+B}}{(s'+1)^{3/2}\sqrt{s'+A}}ds'\right)$$

$$= \delta_{IJ}\frac{(k_I)^{3/2}}{2\sqrt{\det(\boldsymbol{K}_0)}}\frac{2}{A-B}\left(\frac{\mathrm{E}\left(\cos^{-1}(\sqrt{B}),\frac{A-1}{B-1}\right)}{\sqrt{1-B}}-\frac{\mathrm{E}\left(\cos^{-1}(\sqrt{A}),\frac{B-1}{A-1}\right)}{\sqrt{1-A}}\right)$$

where $E(\theta, m) = \int_0^\theta \sqrt{1-m\sin^2\theta'}\,d\theta'$ is an elliptic integral of 2nd kind.

When $A = B$, the only remaining anisotropy factor of the matrix is $A = k_1/k_2$. Then, we have

$$S_{11} = \frac{A}{2}\int_0^\infty \frac{1}{(s'+1)^{\frac{3}{2}}(s'+A)}ds' = A\cdot\left(\frac{1}{A-1}-\frac{\sec^{-1}\sqrt{A}}{(A-1)^{3/2}}\right)$$

and

$$S_{22} = \frac{1}{2\sqrt{A}}\int_0^\infty \frac{1}{(s'+1)^2\sqrt{s'+\frac{1}{A}}}ds' = \frac{A}{2}\cdot\left(\frac{\sec^{-1}\sqrt{A}}{(A-1)^{1.5}}-\frac{1}{\left(A\cdot(A-1)\right)}\right)$$

We note that under the results of the assumption of $A \neq B$, by taking the limit such that $B \rightarrow A$, we get the same result obtained from the assumption of $A = B$. We also note that, for the

$A = B$ case, in the limit of $k_1 \rightarrow k_2 (A \rightarrow 1)$, the Eshelby tensor reduces to the isotropic matrix result, $S_{ij} = \frac{1}{3}\delta_{ij}$. The two independent values (S_{11}, $S_{22} = S_{33}$) are plotted in terms of k_1/k_2 in the Fig. S1(d) where we validate our solutions against the numerical evaluation of Eq. (4).

Supplementary Note 4: Non-uniform heat flux within ellipsoidal inclusion having Kapitza's thermal resistance

We simulate a single inhomogeneity problem with an ellipsoidal inclusion which has an aspect ratio of 2. The length of the cube edge is 10 m and the axis lengths of the inclusion are 1 m and 2 m respectively. The material properties used in this simulation are 1 W/mK (matrix) and 10 W/mK (inclusion). For the case of zero interfacial thermal resistance, the heat flux within the ellipsoidal inclusion is uniform (see Fig. S2). However, the heat flux is not uniform in the presence of an interfacial thermal resistance (see Fig. S2). The heat flux within the inclusion has a maximum at the centre of the inclusion and it decreases as the interfacial thermal resistance increases.

To explain the non-uniformity of the heat flux, we numerically calculate M_{1111} for the ellipsoidal inclusion, assuming uniform heat flux within the inclusion. M_{1111} is uniform when the shape of inclusion is a sphere, so the assumption is reasonable. However, when the inclusion shape is ellipsoidal, the value depends on the position in the inclusion, which means that the assumption cannot be used for the ellipsoidal inclusion case (see Fig. S3). Since the heat flux within the ellipsoidal inclusion is not uniform when interfacial thermal resistance exists, the conventional method cannot be used to calculate the localization tensor and effective modulus analytically

Supplementary Note 5: Proof for Eq. (17)

After using the divergence theorem at Eq. (15), we have

$$M_{ijms}(\mathbf{x}) = \frac{1}{R}\left[\frac{\partial N_{ijs}(\mathbf{x})}{\partial x_m} + \delta_{is} D_{mj}(\mathbf{x})\right],$$

where

$$N_{ijs}(\mathbf{x}) := \int_V \frac{\partial^2 G(\mathbf{y}-\mathbf{x})}{\partial y_i \partial y_j} y_s d\mathbf{y}$$

and

$$D_{mj}(\boldsymbol{x}) := \frac{\partial}{\partial x_m}\int_V \frac{\partial}{\partial y_j} G(\mathbf{y}-\mathbf{x}) d\mathbf{y}$$

with

$$G(\mathbf{x}-\mathbf{y}) := \frac{1}{4\pi\sqrt{\det(\boldsymbol{K}_0)[(\mathbf{x}-\mathbf{y})^T \boldsymbol{K}_0^{-1}(\mathbf{x}-\mathbf{y})]}}.$$

Since

$$\frac{\partial G(\mathbf{x}-\mathbf{y})}{\partial x_i} = -\frac{\partial G(\mathbf{x}-\mathbf{y})}{\partial y_i},$$

we get

$$N_{ijs}(\mathbf{x}) = \int_V \frac{\partial^2 G(\mathbf{y}-\mathbf{x})}{\partial y_i \partial y_j} y_s d\mathbf{y} = \frac{\partial^2}{\partial x_i \partial x_j}\int_V G(\mathbf{y}-\mathbf{x}) y_s d\mathbf{y}\ .$$

Our goal is to show

$$(K_0)_{ij}\frac{\partial N_{ijs}(\mathbf{x})}{\partial x_m} = \delta_{ms}.$$

Noting

$$K_0 = \mathrm{diag}(k_1, k_2, k_3),$$

we rewrite $G(\mathbf{y}-\mathbf{x})$ such that

$$G(\mathbf{y}-\mathbf{x}) = \frac{1}{4\pi\sqrt{k_1 k_2 k_3}} \cdot \frac{1}{\sqrt{\sum_{\ell=1}^{3}\frac{(x_\ell - y_\ell)^2}{k_\ell}}}.$$

This implies that

$$\frac{\partial N_{ijs}(\mathbf{x})}{\partial x_m} = \frac{\partial^3}{\partial x_m \partial x_i \partial x_j}\int_V G(\mathbf{y}-\mathbf{x}) y_s d\mathbf{y}$$

$$= \frac{1}{4\pi\sqrt{k_1 k_2 k_3}}\frac{\partial^3}{\partial x_m \partial x_i \partial x_j}\int_V \frac{y_s}{\sqrt{\sum_{\ell=1}^{3}\frac{(x_\ell - y_\ell)^2}{k_\ell}}} d\mathbf{y}.$$

Now we can get the following by substituting $\tilde{x}_\ell = \frac{x_\ell}{\sqrt{k_\ell}}$ and $\tilde{y}_\ell = \frac{y_\ell}{\sqrt{k_\ell}}$:

$$
\begin{aligned}
&\frac{1}{4\pi\sqrt{k_1k_2k_3}}\frac{\partial^3}{\partial x_m\partial x_i\partial x_j}\int_V \frac{y_S}{\sqrt{\sum_{\ell=1}^3\frac{(x_\ell-y_\ell)^2}{k_\ell}}}d\mathbf{y}\\
&=\frac{1}{4\pi\sqrt{k_1k_2k_3}}\sqrt{k_S}\frac{1}{\sqrt{k_Mk_Ik_J}}\ \frac{\partial^3}{\partial\tilde{x}_M\partial\tilde{x}_I\partial\tilde{x}_J}\int_V \frac{\tilde{y}_S}{\sqrt{\sum_{\ell=1}^3(\tilde{x}_\ell-\tilde{y}_\ell)^2}}\cdot\frac{\partial\mathbf{y}}{\partial\tilde{\mathbf{y}}}d\tilde{\mathbf{y}}\\
&=\frac{1}{4\pi\sqrt{k_1k_2k_3}}\frac{\sqrt{k_S}}{\sqrt{k_Mk_Ik_J}}\frac{\partial^3}{\partial\tilde{x}_M\partial\tilde{x}_I\partial\tilde{x}_J}\int_{\tilde{\omega}} \frac{\tilde{y}_S}{\sqrt{\sum_{\ell=1}^3(\tilde{x}_\ell-\tilde{y}_\ell)^2}}\sqrt{k_1k_2k_3}d\tilde{\mathbf{y}}\\
&=\frac{\sqrt{k_S}}{4\pi\sqrt{k_Mk_Ik_J}}\frac{\partial^3}{\partial\tilde{x}_M\partial\tilde{x}_I\partial\tilde{x}_J}\int_{\tilde{\omega}} \frac{\tilde{y}_S}{\sqrt{\sum_{\ell=1}^3(\tilde{x}_\ell-\tilde{y}_\ell)^2}}d\tilde{\mathbf{y}}.
\end{aligned}
$$

This gives us that

$$
\begin{aligned}
&\sum_{I,J}(K_0)_{IJ}\frac{\sqrt{k_S}}{4\pi\sqrt{k_Mk_Ik_J}}\frac{\partial^3}{\partial\tilde{x}_M\partial\tilde{x}_I\partial\tilde{x}_J}\int_{\tilde{\omega}} \frac{\tilde{y}_S}{\sqrt{\sum_{\ell=1}^3(\tilde{x}_\ell-\tilde{y}_\ell)^2}}d\tilde{\mathbf{y}}\\
&=\sum_{I,J}k_I\delta_{IJ}\frac{\sqrt{k_S}}{4\pi\sqrt{k_Mk_Ik_J}}\frac{\partial^3}{\partial\tilde{x}_M\partial\tilde{x}_I\partial\tilde{x}_J}\int_{\tilde{\omega}} \frac{\tilde{y}_S}{\sqrt{\sum_{\ell=1}^3(\tilde{x}_\ell-\tilde{y}_\ell)^2}}d\tilde{\mathbf{y}}\\
&=\sum_{I}\frac{\sqrt{k_S}}{4\pi\sqrt{k_M}}\frac{\partial^3}{\partial\tilde{x}_M(\partial\tilde{x}_I)^2}\int_{\tilde{\omega}} \frac{\tilde{y}_S}{\sqrt{\sum_{\ell=1}^3(\tilde{x}_\ell-\tilde{y}_\ell)^2}}d\tilde{\mathbf{y}}\\
&=\sqrt{\frac{k_S}{k_M}}\frac{\partial}{\partial\tilde{x}_M}\int_{\tilde{\omega}} \Delta_{\tilde{x}}\frac{1}{4\pi\sqrt{\sum_{\ell=1}^3(\tilde{x}_\ell-\tilde{y}_\ell)^2}}\tilde{y}_Sd\tilde{\mathbf{y}}\\
&=-\sqrt{\frac{k_S}{k_M}}\frac{\partial}{\partial\tilde{x}_M}\int_{\tilde{\omega}}\delta(\tilde{x}-\tilde{y})\tilde{y}_Sd\tilde{\mathbf{y}}\\
&=-\sqrt{\frac{k_S}{k_M}}\frac{\partial\tilde{x}_S}{\partial\tilde{x}_M}\\
&=-\delta_{ms}.
\end{aligned}
$$

Hence, our goal is proven.

Here the capital index has the same value with small index and the repeated capital index are not summed over.

From the definition of Eshelby tensor (Eq. 4),

$$D_{mj}(\boldsymbol{x})K_{0_{ij}} = \frac{\partial}{\partial x_j}\int_V \frac{\partial G(\boldsymbol{x}-\boldsymbol{y})}{\partial y_m} d\boldsymbol{y}\, K_{0_{ij}} = S_{im}$$

So,

$$K_{0_{ij}} M_{ijms}(\mathbf{x}) = \frac{1}{R} K_{0_{ij}} \left[\frac{\partial N_{ijs}(\mathbf{x})}{\partial x_m} + \delta_{is} D_{mj}(\mathbf{x})\right] = \frac{1}{R}(-\delta_{ms} + S_{ms})$$

which is equivalent to

$$\boldsymbol{K}_0 : \boldsymbol{M} = \frac{1}{R}(-\boldsymbol{I} + \boldsymbol{S}).$$

References

1 Giordano, S. & Palla, P. L. Dielectric behavior of anisotropic inhomogeneities: interior and exterior point Eshelby tensors. *Journal of Physics A: Mathematical and Theoretical* **41**, 415205, doi:10.1088/1751-8113/41/41/415205 (2008).

2 Mathematica, W. Wolfram Research. *Inc., Champaign, Illinois* (2009).
